# Positron Emission Tomography Reveals Abnormal Topological Organization in Functional Brain Network in Diabetic Patients

**DOI:** 10.3389/fnins.2016.00235

**Published:** 2016-05-27

**Authors:** Xiangzhe Qiu, Yanjun Zhang, Hongbo Feng, Donglang Jiang

**Affiliations:** Department of Nuclear Medicine, The First Affiliated Hospital of Dalian Medical UniversityDalian, China

**Keywords:** PET, FDG, diabetes, network, graph theory, small world

## Abstract

Recent studies have demonstrated alterations in the topological organization of structural brain networks in diabetes mellitus (DM). However, the DM-related changes in the topological properties in functional brain networks are unexplored so far. We therefore used fluoro-D-glucose positron emission tomography (FDG-PET) data to construct functional brain networks of 73 DM patients and 91 sex- and age-matched normal controls (NCs), followed by a graph theoretical analysis. We found that both DM patients and NCs had a small-world topology in functional brain network. In comparison to the NC group, the DM group was found to have significantly lower small-world index, lower normalized clustering coefficients and higher normalized characteristic path length. Moreover, for diabetic patients, the nodal centrality was significantly reduced in the right rectus, the right cuneus, the left middle occipital gyrus, and the left postcentral gyrus, and it was significantly increased in the orbitofrontal region of the left middle frontal gyrus, the left olfactory region, and the right paracentral lobule. Our results demonstrated that the diabetic brain was associated with disrupted topological organization in the functional PET network, thus providing functional evidence for the abnormalities of brain networks in DM.

## Introduction

Diabetes mellitus (DM) has emerged as an important risk factor for cognitive dysfunction and dementia. The observed impairments in cognitive function are hypothesized to be subserved by alterations structure and function in diabetic brain (Mansur et al., 2014). Evidence from previous neuroimaging studies has shown DM-related brain abnormalities, involving global and regional atrophy in frontal, temporal and posterior cortex (Den Heijer et al., 2003; van Elderen et al., 2010; Frøkjaer et al., 2013; Hughes et al., 2013), decreased neuronal activities predominantly in prefrontal and middle temporal gyrus correlated with cognitive dysfunctions (Xia W. et al., 2013; García-Casares et al., 2014a,b), as well as alterations in structural and functional connectivity between different regions (Musen et al., 2012; van Duinkerken et al., 2012; Antenor-Dorsey et al., 2013; Reijmer et al., 2013a; Chen et al., 2015). Recently, a growing number of studies also have provided significant insights into the abnormalities in the functional integration of the entire brain networks (Lyoo et al., 2013; Reijmer et al., 2013b; van Duinkerken et al., 2016). For example, by using cortical thickness (Lyoo et al., 2013) and gray matter volume (van Duinkerken et al., 2016) from magnetic resonance imaging (MRI), studies found that structural brain network was disrupted in type 1 diabetes mellitus (T1DM) patients. Moreover, a study performed diffusion tensor imaging (DTI) and also found damage to the brain white matter network in type 2 diabetes mellitus (T2DM) patients, which was related to slowing of information processing speed (Reijmer et al., 2013b). Network-based measures can be more sensitive to alterations that are not apparent in specific regions because they consider each region's integration into the global unit rather than as an independent entity (Brown et al., 2011). Therefore, studies of the brain network using neuroimaging technology may provide a new approach for the diagnosis and treatment of this disease.

As a conventional functional imaging technology, fluoro-D-glucose positron emission tomography (FDG-PET) can reflect brain activity by detecting changes in the brain glucose metabolism (Sokoloff, 1981; Jueptner and Weiller, 1995), which is different from other types of imaging modality. With respect to DM, several studies have found reduced cerebral glucose metabolism primarily in frontal an temporal regions (Baker et al., 2011; García-Casares et al., 2014a,b; Roberts et al., 2014). Meanwhile, previous studies have suggested that inter-subject correlations of metabolic rates are capable of reflecting functional relationships between regions (Horwitz et al., 1984, 1987; Di et al., 2012; Morbelli et al., 2013). And on this basis, recent studies have constructed functional networks of the human brain from FDG-PET data and further characterized by using graph theoretical approaches (Sanabria-Diaz et al., 2013; Seo et al., 2013; Kim et al., 2015; Yao et al., 2015). Through these approaches, metabolic networks of human brain have been consistently found to have a “small-world” topology, characterized by a high cluster coefficient like a regular network and small path length like a random network (Watts et al., 1998).

Given the existence of metabolic abnormalities in specific brain regions (Baker et al., 2011; Roberts et al., 2014) as well as disruption of structural brain networks in DM patients, it is plausible that the abnormalities of whole brain functional network in DM may be observed by using FDG-PET technique. However, to our knowledge, the DM-related changes in the topological properties in functional network based on FDG-PET data are unexplored so far. Thus, we used graph theoretical analysis and FDG-PET technique to investigate the hypothesis that the topological properties of brain functional network in DM is disrupted. First, we assessed the small-world topology of each network. Second, we investigated the topological parameters of the functional networks (small-world index, normalized clustering coefficient, and normalized characteristic path length) at different connection densities. Finally, we observed the hub regions and evaluated the statistical differences in nodal centrality between the DM and NC groups.

## Data and methods

### Subjects

This was a retrospective analysis and all subjects were selected from a data pool in the PET Center of the First Affiliated Hospital of Dalian Medical University. A total of 73 diabetic patients (54 males, 19 females) with a mean age of 57 ± 10 years, and 91 normal individuals (66 males, 25 females) with a mean age of 56 ± 8 years, were selected into this study (Table 1). The subjects met the following criteria: (1) All diabetic patients met the diagnosis proposed by the World Health Organization 1999 (Alberti and Zimmet, 1998) and these patients had peripheral blood glucose levels of >7.0 mmol/L; (2) Most of the normal individuals in the normal control group were individuals recruited during routine health checks. They had no history of diabetes, and their peripheral blood glucose levels were 4.1–5.5 mmol/L; (3) All subjects had no metabolic diseases such as hyperthyroidism or hypothyroidism; (4) All subjects had no prior stroke, transient ischemic attack, epilepsy, headache, brain trauma, mental illness, or carbon monoxide poisoning; (5) All subjects had no severe heart, liver, spleen, pancreas, or kidney diseases; (6) All subjects had no history of heavy drinking; (7) All subjects had no estrogen replacement therapy for postmenopausal women; (8) All subjects had no prior chemotherapy or radiotherapy in the head or neck. Additionally, this study was approved by the Institutional Review Board of the First Affiliated Hospital of Dalian Medical University.

**Table 1:** **Demographic and clinical data of the subjects**.

	**DM group (*n* = 73)**	**NC group (*n* = 91)**	***P***
Gender (male)	54	66	0.84
Age (years)	57 ± 10	56 ± 8	0.25
Fasting blood glucose (mmol/L)	8.4 ± 0.9	5.1 ± 0.4	< 0.001
Duration of illness (years)	8.0 ± 6.6	–	–

### Acquisition of PET data

The Eclipse RD cyclotron (Siemens AG, Germany) and FDG synthesis device (Siemens ExploraFDG4, Germany) were used to synthesize 18F-FDG, and the resulting radiochemical purity was >95%. All of the subjects were asked to fast at least 6 h before the exam, rested for 15 min in a dark, quiet environment, and received intravenous FDG 0.15 mCi/kg via the cubital vein. Afterward, the subjects continued to rest for 40–50 min and then underwent head PET scans for 3 min. The PET scanner used was a Siemens Biograph 64 HD PET/CT (Siemens AG, Germany), and the spatial resolution of the scanner is 4.2 mm full width at half maximum (FWHM) in axial, sagittal or coronal plane. The PET scan used the 3-dimensional (3D) mode.

### Data preprocessing

Image format conversion, spatial normalization, and smoothing were performed during data processing using the MATLAB platform-based SPM8 software. Spatial normalization was performed using the standard template of the Montreal Neurological Institute, and smoothing was performed using an isotropic Gaussian kernel with an 8 mm full width at half maximum to increase the signal-to-noise ratio (Liu et al., 2014).

### Construction of brain functional PET network

In graph theory analysis, the network consists of many nodes and edges that connect the nodes (Rubinov and Sporns, 2010). In this study, we employed anatomically automatic labeled (AAL) to divide the cortex into 90 regions of interest (ROIs; 45 in each hemisphere except cerebellum) represented nodes, which has been broadly used in many structural and functional brain network studies (Yan et al., 2011; Sanabria-Diaz et al., 2013; Wang et al., 2014). The names of the ROIs and their abbreviations are listed in Table 2. The mean glucose metabolism value of each ROI was used to construct functional network. Before performing a correlation analysis, we normalized the mean glucose metabolism value of each ROI (i.e., we divided the mean glucose metabolism value of each ROI by that of the whole brain), and we then used a linear regression model to control for age, gender, and the effect of blood glucose on the local glucose metabolism. The resulting regression residual was used to substitute the local glucose metabolism value for a Pearson correlation analysis to construct the inter-regional correlation matrix for glucose metabolism. It is also noteworthy that the correlation was calculated across subjects in each group and one network was obtained for each group. This method has been employed in other functional brain network studies (Sanabria-Diaz et al., 2013; Liu et al., 2014).

**Table 2:** **Anatomical parcellation defined by automated anatomical labeling atlas and abbreviations for the regions of interest (ROIs)**.

**Abbreviations**	**Regions**
PreCG	Precental gyrus
SFGdor	Superior frontal gyrus, dorsolateral
ORBsup	Superior frontal gyrus, orbital part
MFG	Middle frontal gyrus
ORBmid	Middle frontal gyrus, orbital part
IFGoperc	Inferior frontal gyrus, opercular part
IFGtriang	Inferior frontal gyrus, triangular part
ORBinf	Inferior frontal gyrus, orbital part
ROL	Rolandic operculum
SMA	Supplementary motor area
OLF	Olfactory cortex
SFGmed	Superior frontal gyrus, medial
ORBsupmed	Superior frontal gyrus, medial orbital
REC	Gyrus rectus
INS	Insula
ACG	Anterior cingulate and paracingulate gyri
DCG	Median cingulate and paracingulate gyri
PCG	Posterior cingulate gyrus
HIP	Hippocampus
PHG	Parahippocampal gyrus
AMYG	Amygdala
CAL	Calcarine fissure and surrounding cortex
CUN	Cuneus
LING	Lingual gyrus
SOG	Superior occipital gyrus
MOG	Middle occipital gyrus
IOG	Inferior occipital gyrus
FFG	Fusiform gyrus
PoCG	Postcentral gyrus
SPG	Superior parietal gyrus
IPL	Inferior parietal, but supramarginal and angular gyri
SMG	Supramarginal gyrus
ANG	Angular gyrus
PCUN	Precuneus
PCL	Paracentral lobule
CAU	Caudate nucleus
PUT	Lenticular nucleus, putamen
PAL	Lenticular nucleus, pallidum
THA	Thalamus
HES	Heschl gyrus
STG	Superior temporal gyrus
TPOsup	Temporal pole: superior temporal gyrus
MTG	Middle temporal gyrus
TPOmid	Temporal pole: middle temporal gyrus
ITG	Inferior temporal gyrus

*The listed names are for one hemisphere*.

### Graph theory analysis of brain functional PET network

We converted the inter-regional correlation coefficient matrix into a binary matrix: in the 90 × 90 correlation coefficient matrix, if the absolute value of the correlation coefficient was above a certain threshold, there was a connection (assigned “1”); otherwise, there was no connection (assigned “0”; Liu et al., 2014; Kim et al., 2015). Currently, researchers have no consensus about how to choose a fixed threshold, we therefore threshold each correlation matrix over a wide range of density (10–40% with a 1% increment), then estimated the properties of the resulting graphs at each threshold value. In present study, the lowest density where the largest component size was 90 was density 10%. The range of density bigger than 10% also ensured that every nodal pairs in both graphs had a connecting path and minimizing the number of false-positive paths (Liu et al., 2014). It also can characterize the network with continuous weighting between nodes, but this will lead to complicated descriptions of statistical features in graph theoretical analysis (He et al., 2007). Therefore, this study used binarized networks to a relatively simpler statistical features descriptions.

#### Cluster coefficient

The parameter cluster coefficient *C* reflects the extent of network clustering (Watts et al., 1998; Sporns et al., 2004). The value of the cluster coefficient *C*_*i*_ of node *i* was the ratio of the number of actual connecting edges *e*_*i*_ between the node and neighboring nodes to the maximum possible number of connecting edges. The mean cluster coefficient of all the nodes in the network was the cluster coefficient *C* of the network, which was calculated using the following formula:

(1) $$\begin{array}{l} C=\frac{1}{\text{N}}\sum C_{i},\;C_{i}=\frac{2e_{i}}{k_{i}\left(k_{i}-1\right)} \end{array}$$

Note: *k*_*i*_ is the number of neighboring nodes of node *i*.

#### Characteristic path length

The shortest path length depicted the optimal path for the information in one node to reach another node in the network. Information was transmitted faster via the shortest path, thus saving system resources (Latora and Marchiori, 2001). The path between nodes *i* and *j* with the smallest number of edges was the shortest path between two nodes, and the number of edges in the path was the shortest path length L_*ij*_ between nodes i and *j*. The characteristic path length of the network described the mean shortest path length between any two nodes of the network, which was calculated using the following formula:

(2) $$\begin{array}{l} \text{L}=\frac{1}{\text{N}\left(\text{N}-1\right)}\sum {\text{L}}_{ij} \end{array}$$

#### “Small-world” network

Studies have shown that regular networks have a larger cluster coefficient and a longer characteristic path length, while random networks have a smaller cluster coefficient and a shorter characteristic path length (Watts et al., 1998). In-between networks have a relatively large cluster coefficient similar to that of regular networks, and a relatively short characteristic path length similar to that of random networks. In other words, these in-between networks combine the benefits of regular networks and random networks, thus ensuring the efficiency of local and global information transmission. Therefore, these networks with a large cluster coefficient and a short characteristic path length are known as “small-world” networks. To quantify the “small-world” characteristics of these networks, a random network was used as a reference. If the network being studied has a larger cluster coefficient and an approximate shortest path length relative to the random network (normalized clustering coefficient $\gamma =\frac{C_{real}}{C_{random}}\gg 1$, normalized characteristic path length $\lambda \;=\frac{L_{real}}{L_{random}}\sim 1\;$ where the subscript “random” represents a random network and “real” represents a real network), then the network is a “small-world” network. Some researchers have proposed unifying the two metrics into one (σ), and the small-world index σ = γ/λ is used to measure the “small-world” characteristics of the network (Achard, 2006; Humphries et al., 2006). The random network and the corresponding network have the same number of nodes, average degree and degree distribution (Sanabria-Diaz et al., 2010).

#### Betweenness centrality

The betweenness centrality defines the centrality of nodes from the perspective of information flow (Freeman, 1977). For any node *i* in the network, its betweenness centrality B(*i*) was calculated with the following formula:

(3) $$\begin{array}{l} \text{B}\left(i\right)=\frac{\sum n_{jk}\left(\text{i}\right)}{n_{jk}} \end{array}$$

where *n*_*jk*_ is the number of all shortest paths from node *j* to node *k*, and *n*_*jk*_(*i*) is the number of shortest paths that go through node *i*. We used *b*_*i*_ = B(*i*)/B to normalize B(i), where B represents the mean betweenness centrality of all the nodes in the network. When normalized betweenness (*b*_*i*_) is >1.5, node *i* represents the hub region in the network (Melie-García et al., 2013).

In this study, both the global and nodal parameters were computed by using software packages of GRETNA (Wang et al., 2015).

### Statistical analysis

1. Statistics of the differences in gender, age and blood glucose between the two groups: A two-sample *T*-test was performed to analyze the difference in age and blood glucose between the two groups, and a chi-square test was performed to analyze the difference in gender between the two groups. No significant difference in gender and age between the two groups was detected (Table 1).
2. Statistics of the difference between the network parameters: We used a non-parametric test to determine whether the difference in the brain network parameters was significant between the diabetic group and the normal group. (1) At different density thresholds, we calculated the normalized cluster coefficient γ, the normalized characteristic path length λ, the small-world index σ, and the betweenness centrality B(*i*) of each group; (2) assuming that the difference in each network parameter between the two groups was due to randomization, we randomly assigned the two groups of subjects again to form two new groups, and we calculated the correlation coefficient matrix, re-constructed two brain networks, and calculated each parameter for the two networks using different density thresholds; (3) we repeated the randomization, constructed new networks, and calculated the network parameters 1000 times to obtain the distribution of the inter-group differences in the network parameters under different thresholds; (4) we used the 95 percentile points of the distribution as the critical values for one-tailed to analyze whether the null hypothesis with a probability of type I error of 0.05. If the null hypothesis was rejected, then the inter-group difference in the brain network parameters was significant (He et al., 2008).

## Results

### Changed cerebral glucose metabolism in DM patients

**Figure 1:**
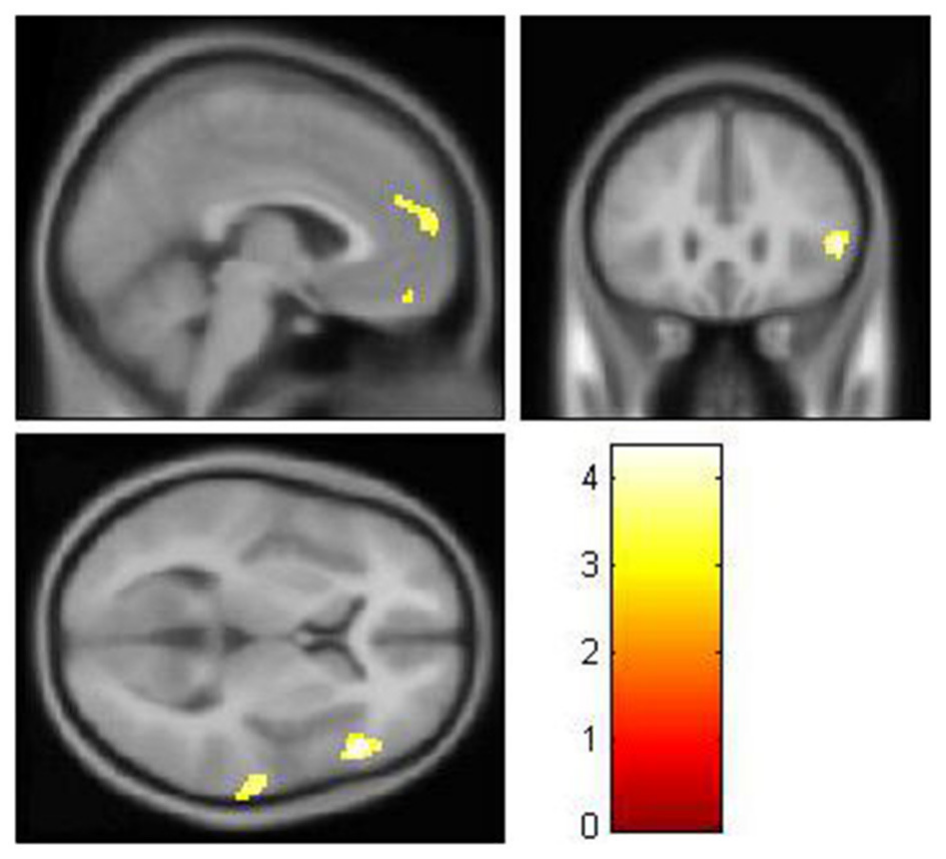
**The decreased glucose metabolism regions in DM (***P*** < 0.001, uncorrected)**. The cluster size >50 voxels. The color bar indicates the *T*-value. Small-world topology of the functional networksThe brain functional networks of both the DM group and the NC group had the characteristics of “small-world” networks (σ > 1). For example, at all of the density thresholds, both networks had a large normalized cluster coefficient (γ >> 1) and a normalized characteristic path length close to 1 (λ ~ 1; Figure 2).

After controlling for the effect of age, gender, and glucose level, the local brain glucose metabolism rate of the DM group was significantly lower than that of the NC group (Table 3, Figure 1), and there were no significant higher metabolism regions in DM group compared with NC group (*P* < 0.001, uncorrected). Overall, the DM group presented reduced cerebral glucose metabolism in several frontal and temporal brain areas, which was coherent with the previous studies (Baker et al., 2011; García-Casares et al., 2014a,b; Roberts et al., 2014).

**Table 3:** **The decreased glucose metabolism regions in DM (***P*** < 0.001, uncorrected)**.

**AAL regions**	**Volume (voxels)**	***T***	**Coordinates**			**BA**
			**x**	**y**	**z**	
Superior frontal gyrus, medial (Left)	136	3.7	0	45	26	9
Superior temporal gyrus (Right)	58	3.8	66	−18	4	42
Inferior frontal gyrus, orbital part (Right)	66	4.3	44	40	−6	47
Inferior frontal gyrus, triangular part (Right)	228	4.4	50	28	6	45

*Coordinates x,y,z refer to the anatomical location of peak voxel defined by the Montreal Neurological Institute space. All regions significant on voxel level uncorrected P < 0.001. BA indicates Brodmann's area*.

### Small-world topology of the functional networks

**Figure 2:**
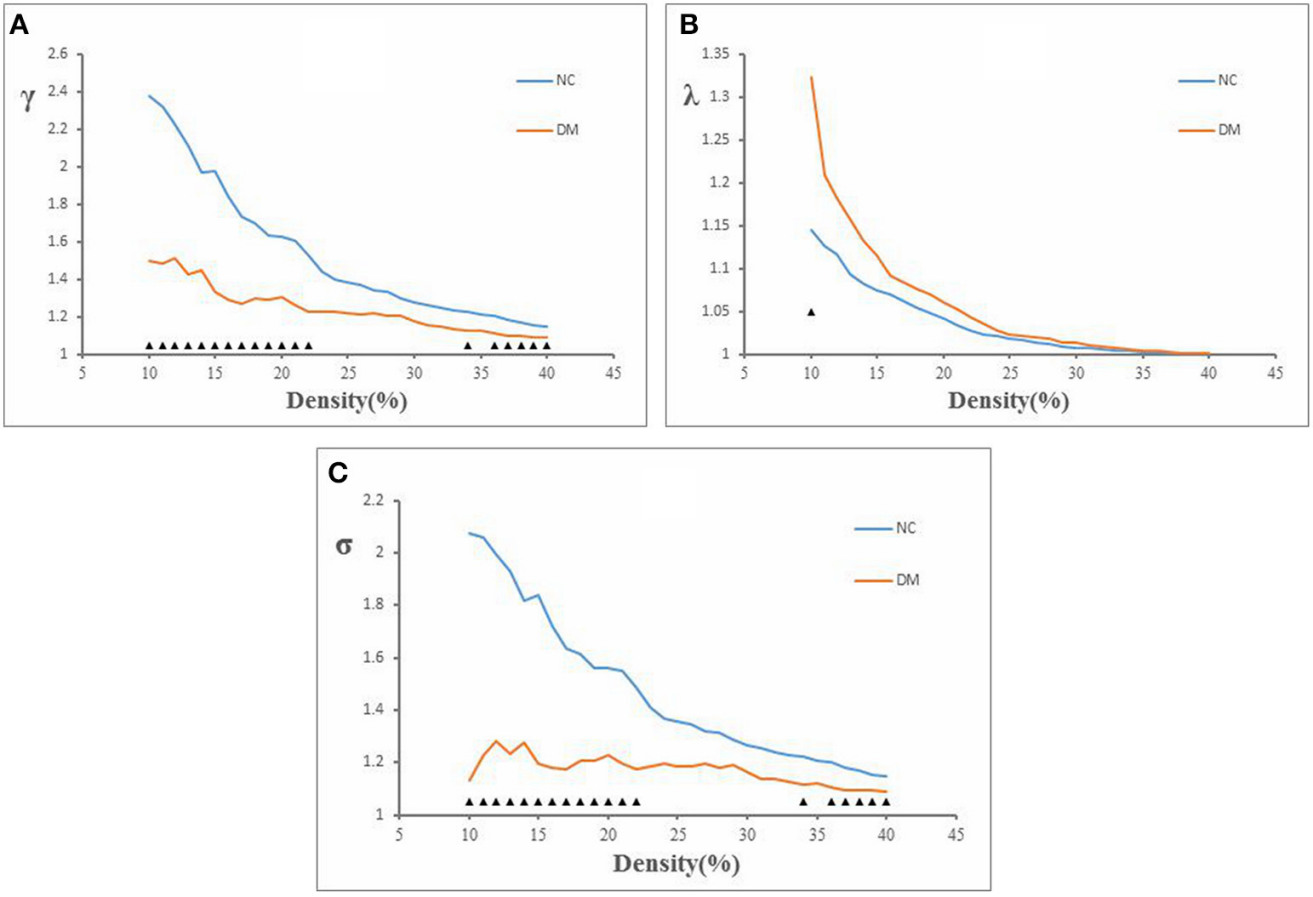
**Normalized cluster coefficient (γ), normalized characteristic path length (λ), and small-world index (σ) in DM and NC. (A)** The γ from NC and DM. *p* < 0.05 for DM vs. NC (significant at 10–23, 34, and 36–40%). **(B)** The λ from NC and DM. *p* < 0.05 for DM vs. NC (significant at 10%). **(C)** The σ from NC and DM. *p* < 0.05 for DM vs. NC (significant at 10–23, 34, and 36–40%).

The brain functional networks of both the DM group and the NC group had the characteristics of “small-world” networks (σ > 1). For example, at all of the density thresholds, both networks had a large normalized cluster coefficient (γ >> 1) and a normalized characteristic path length close to 1 (λ ~ 1; Figure 2).

### Altered small-world parameters in DM patients

This study showed that the normalized clustering coefficient γ was significantly smaller in the DM group than in the NC group (density thresholds: 10–22, 34, 36–40%; Figure 2A), the normalized characteristic path length λ was significantly longer in the DM group than in the NC group (density threshold: 10%; Figure 2B), and the small-world index σ was significantly smaller in the DM group than in the NC group (density thresholds: 10–22, 34, 36–40%; Figure 2C) (*P* < 0.05).

### The distribution of the hub regions and altered nodal centrality in DM patients

To explore the nodal centrality, the functional networks were constructed at a fixed density threshold of 10%, which ensures that all regions were included in the functional networks and minimizing the number of false-positive paths. Furthermore, such a fixed constraint might optimize interregional correlation strengths and therefore be biologically plausible (Bassett and Bullmore, 2006; He et al., 2008).

**Figure 3:**
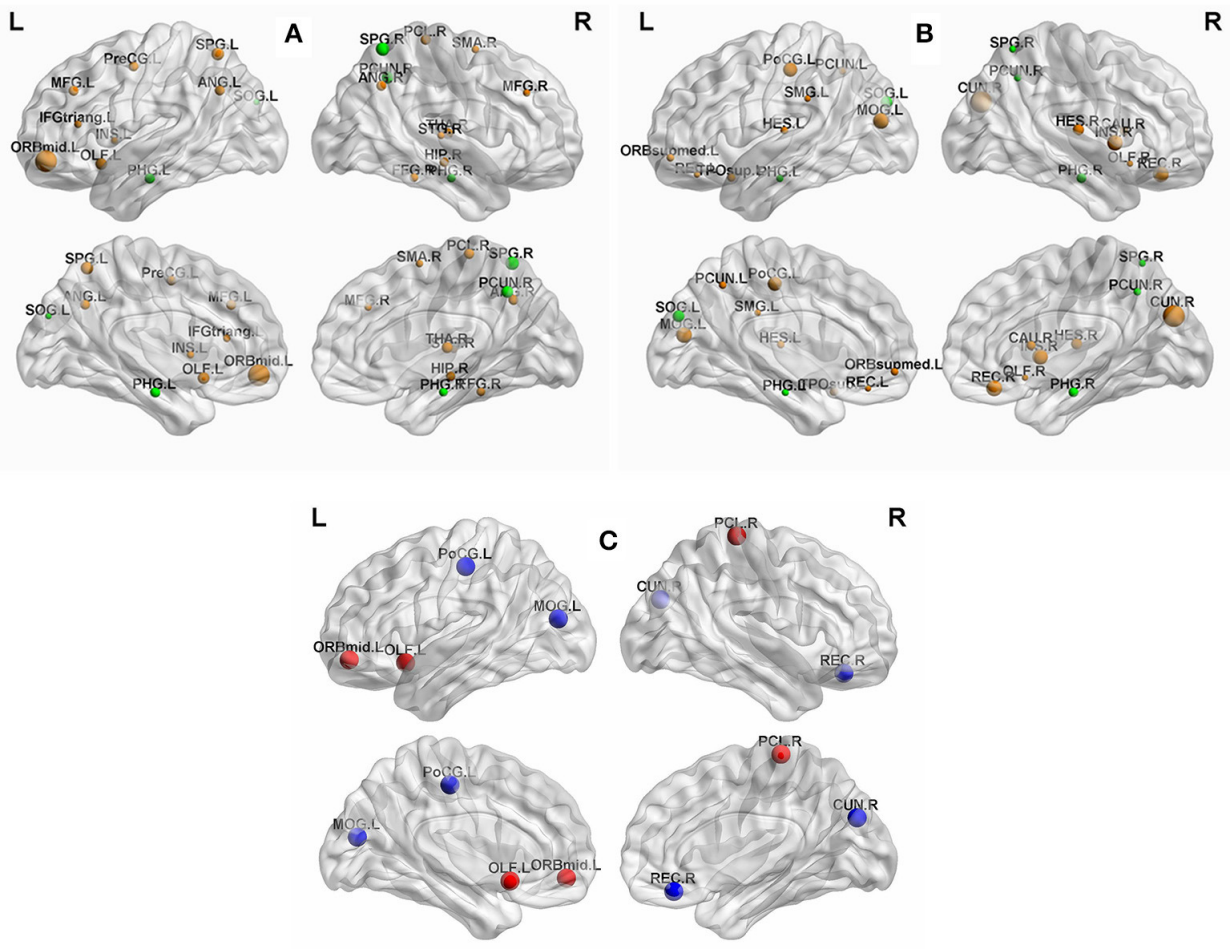
**(A)** The hub regions in DM. **(B)** The hub regions in NC. **(C)** Differences of between-group betweenness centrality in DM and NC (*p* < 0.05). The green spheres are represented the common hub regions. The sphere diameter denotes normalized betweenness centrality (b_*i*_), in this case b_*i*_ > 1.5. The blue spheres represent betweenness centrality with significant decreases (four hub regions) and the red spheres represent betweenness centrality with significant increases (three hub regions) in DM compared with NC. The fixed density threshold value was 10%. Hubs were visualized with the BrainNet Viewer (http://www.nitrc.org/projects/bnv; Xia M. et al., 2013).

To identify the hub regions, we measured the normalized betweenness centrality in both networks (see Section Data and Methods). The DM had 21 hub regions (Table 4), and the NC had 19 (Table 5). The two groups shared five hub regions (Figures 3A,B). We used a 1000 non-parametric permutation test and obtained seven hub regions with significant differences between the DM patients and NCs (*P* < 0.05; Figure 3C). In addition, given that it is no reason to say that there are hub shifts between groups if normalized betweenness centrality distribution are highly correlated between the two groups, we therefore have verified that the normalized betweenness centrality distribution between the two groups are no significantly correlated (*R* = −0.03, *P* = 0.77). Compared with NCs, the centrality was significantly reduced in four brain regions: the right rectus, the right cuneus, the left middle occipital gyrus, and the left postcentral gyrus, and it was significantly increased in three brain regions: the orbitofrontal region of the left middle frontal gyrus, the left olfactory region, and the right paracentral lobule in the diabetic patients.

**Table 4:** **The hubs of functional network in DM group**.

**Region (DM)**	**Classification**	**b*_*i*_***
Middle frontal gyrus, orbital part (Left)	Paralimbic	7.14
**Superior parietal gyrus (Right)**	Association	4.14
Superior parietal gyrus (Left)	Association	3.82
**Precuneus (Right)**	Association	3.55
Olfactory cortex (Left)	Paralimbic	3.47
Thalamus (Right)	Subcortical	3.22
Paracentral lobule (Right)	Association	2.98
**Parahippocampal gyrus (Left)**	Paralimbic	2.98
Angular gyrus (Right)	Association	2.92
Middle frontal gyrus (Left)	Association	2.88
Angular gyrus (Left)	Association	2.85
Precental gyrus (Left)	Paralimbic	2.72
Hippocampus (Right)	Paralimbic	2.64
**Parahippocampal gyrus (Right)**	Paralimbic	2.40
Fusiform gyrus (Right)	Association	2.39
Supplementary motor area (Right)	Association	1.99
Inferior frontal gyrus, triangular part (Left)	Association	1.98
Middle frontal gyrus (Right)	Association	1.95
Insula (Left)	Paralimbic	1.91
Superior temporal gyrus (Right)	Association	1.85
**Superior occipital gyrus (Left)**	Association	1.53

*This table list the hub regions (b_i_ > 1.5) in the functional network of DM group. The common hub regions for both groups are indicated by bold. b_i_ denotes the normalized betweenness centrality of the region i*.

**Table 5:** **The hubs of functional network in NC group**.

**Region (NC)**	**Classification**	**b*_*i*_***
Cuneus (Right)	Association	6.18
Gyrus rectus (Right)	Association	4.42
Middle occipital gyrus (Left)	Association	4.38
Insula (Right)	Paralimbic	4.33
Postcentral gyrus (Left)	Paralimbic	3.92
**Superior occipital gyrus (Left)**	Association	3.21
Heschl gyrus (Right)	Paralimbic	3.02
**Parahippocampal gyrus (Right)**	Paralimbic	2.61
Caudate nucleus (Right)	Subcortical	2.40
Temporal pole: superior temporal gyrus (Left)	Paralimbic	2.15
Superior frontal gyrus, medial orbital (Left)	Paralimbic	2.04
Heschl gyrus (Left)	Paralimbic	2.04
Precuneus (Left)	Association	1.95
**Precuneus (Right)**	Association	1.94
**Parahippocampal gyrus (Left)**	Paralimbic	1.85
**Superior parietal gyrus (Right)**	Association	1.82
Supramarginal gyrus (Left)	Association	1.69
Gyrus rectus (Left)	Association	1.61
Olfactory cortex (Right)	Paralimbic	1.57

*This table list the hub regions (b_i_ > 1.5) in the functional network of NC group. The common hub regions for both groups are indicated by bold. b_i_ denotes the normalized betweenness centrality of the region i*.

## Discussion

Because the local glucose metabolism rate is a possible contributing factor in DM-related functional network, we first showed the reduced glucose metabolism rate in DM group, which is similar to previous findings. Then we focused to demonstrate alterations in the topological organization of functional brain networks in DM patient by using FDG-PET data. Our main findings included the following: (1) Both DM patients and NCs had a small-world topology in functional brain network; (2) The DM group was found to have significantly lower small-world index, lower normalized clustering coefficients, and higher normalized characteristic path length compared with NC group; (3) The spatial distribution of hub regions and nodal centrality were changed in DM group.

### Small-world properties of the functional networks

Our results showed that brain functional network of both DM patients and NCs had a small-world properties, which was consistent with many studies of brain structural and functional network constructed from MRI, EEG, and FDG-PET data (He et al., 2008; de Haan et al., 2009; Lo et al., 2010; Sanz-Arigita et al., 2010; Sanabria-Diaz et al., 2013; Liu et al., 2014; Kim et al., 2015). In particular, other PET studies have reported that the human brain functional PET networks of both normal individuals and patients with certain diseases (such as AD and MCI) fit the characteristics of small-world network (Sanabria-Diaz et al., 2013; Seo et al., 2013; Hu et al., 2014; Kim et al., 2015). Unlike regular network or random network, a small-world network is an optimized network for information separation and integration (Bullmore and Sporns, 2009). In this study, the brain functional network of diabetic patients also showed small-world property, suggesting that even in human brains afflicted with certain diseases (such as AD and MCI in other studies), a relatively efficient network was maintained in order to meet the needs of daily activities (Zhao et al., 2012).

### Changed in the small-world parameters in DM patients

Although functional PET network in diabetic patients preserved small-world characteristics, several network parameters (γ, λ, σ) were found to be significantly changed. We showed that DM group had significantly lower normalized clustering coefficients and higher normalized characteristic path length as compared with NC group (Figure 2). The cluster coefficient reflected the local efficiency and fault tolerance of the network (Strogatz, 2001). The normalized clustering coefficients of brain functional network was found to be lower in DM patients, indicating that these patients have a reduced ability for local information processing and a lower processing efficiency. Given that short path length ensures the effective integration and prompt transfers of information between distant brain areas (Sporns and Zwi, 2004), our finding of increased normalized characteristic path length in DM network indicated a slower inter-regional information integration and long-distance information transfer in diabetic patients. Our result in functional brain networks were conformed to a recent study about structural brain networks by using DTI data, in which the diabetic group showed significantly lower clustering coefficient and higher characteristic path length compared with control group (Reijmer et al., 2013b). The similar finding in structural and functional brain network analysis may suggest that DM-related alterations in cortical functional networks can be related to structural impairments. Additionally, DM has emerged as an important risk factor for cognitive dysfunction (McCrimmon et al., 2012). Human cognitive $precessing are believed to be built up on a basis of information interactions between interconnected brain regions (Horwitz, 2003; Sporns, 2011). Therefore, the disrupted topological properties in functional network may also contribute to DM-related cognitive impairment.

### The distribution of the hub regions in DM patients and NCS

We also used the betweenness centrality to define important hub regions of the brain network. In this study, if the centrality of a node was 1.5-fold higher than the mean centrality of all of the nodes in the network (Melie-García et al., 2013), the region represented by the node was considered a hub region in the functional network. Figures 3A,B showed hub regions in diabetic patients and normal individuals. We found that 95.2% (20/21) DM hubs (Table 4) and 94.7% (18/19) NC hubs (Table 5) were localized in association and paralimbic regions. Association and paralimbic regions are divided out from the cerebral cortex by using a functional rather than strictly cytoarchitectonic approach (Mesulam, 2000). Studies have shown that association areas help to maintain the integrity of multifunction systems, such as memory and attention, and their function mainly involves intelligence information processing and maintaining advanced mental activities, while paralimbic areas are highly correlated with the prefrontal lobe and many subcortical regions, which are intricately involved in emotions and states of consciousness (Mesulam, 1998; Liu et al., 2014). This result is in line with previous studies that association and paralimbic cortices regions tend to be hubs of brain structural and functional network (He et al., 2008; Tian et al., 2011; Yan et al., 2011; Hu et al., 2014). Meanwhile, five brain regions were identified as the common hubs for both groups, which may indicate that DM poses little impact on these hub regions.

### Altered nodal centrality in DM patients

Our analysis showed that the regions with significant reduced centrality were located in the right rectus, the right cuneus, the left middle occipital gyrus, and the left postcentral gyrus in diabetic patients (*P* < 0.05; Figure 3C, blue sphere area), all of which in present study were found as hub regions in NC group. These hub regions have been reported to be related to DM outcomes in previous neuroimaging studies of the brain structure and function (Cui et al., 2014; Marzelli et al., 2014; Zhang et al., 2014). Cui et al. showed, in their fMRI study, that neural activity was reduced in the cuneus, the middle occipital gyrus, and the postcentral gyrus in DM patients, while changes in neural activity in the cuneus and the postcentral gyrus were associated with cognitive impairment (Cui et al., 2014). Zhang et al. found that DM patients, with or without cognitive impairment, had atrophy of the middle occipital gyrus (Zhang et al., 2014). Matthew et al. found that in DM patients, rectus atrophy was associated with the disease course of diabetes (Marzelli et al., 2014). Several other studies have also reported that disruption of these regions involved in many essential functions were associated with behavioral and cognitive brain functioning. The cuneus is involved in basic visual information processing (Beason-Held et al., 1998; Slotnick and Schacter, 2006). Neural abnormalities in the cuneus were thought to have contributed to poor performance on related cognitive tests (Cui et al., 2014). The middle occipital gyrus is the secondary visual cortex that is involved in the visual spatial and visual perceptive functions (Schurz et al., 2013; Tu et al., 2013), while the postcentral gyrus is part of the primary motor cortex. The decreased of neural activity in the occipital region and postcentral gyrus were found to be associated with visual impairment (Xia W. et al., 2013) and sensory loss (Luo et al., 2012), respectively. The rectus was thought to be part of a circuit that mediates some specific emotional functions in humans (Andreasen et al., 1995). Thus, the observed features from the network models of brain function were consistent with and supportive of the findings from other researches.

The altered hub regions in NC presented structural and functional abnormalities in DM from the above evidence indicated that DM targets some important regions that could disturb the normal functional network. This finding supports the viewpoint that the high level of centrality of hubs makes them points of vulnerability that are susceptible to dysfunction in brain disorders (van den Heuvel and Sporns, 2013), which may be potential pathological mechanism that disrupts the properties of functional brain network in DM. In addition, it should be noted that betweenness centrality as a local property integrates the global information of whole network. The regions of reduced centrality didn't overlap with that of hypometabolism in DM in present study indicated that the disturbances of hub regions may not be explained entirely by structural and functional abnormalities in specific brain regions. Therefore, the mechanism of the normalization betweenness centrality variation correlated to structural and functional changes is very complicated and deserves a detailed study.

Moreover, our results showed that compared with normal individuals, the nodal centrality was significantly increased in the orbitofrontal region of the left middle frontal gyrus, the left olfactory region, and the right paracentral lobule in DM patients (*P* < 0.05; Figure 3C, red sphere area). The betweenness centrality was defined as the centrality of the node from the perspective of information flow. Thus, the increased in centrality of these hub regions may be a compensatory mechanism for the decrease in centrality of other regions in the process of information exchange (Tijms et al., 2013). That is, the degeneration of some hub regions may also lead to an increased betweenness centrality in other regions (van den Heuvel and Sporns, 2013). To our knowledge, this study was the first base on FDG-PET data to identify hub regions with significant differences in the betweenness centrality between DM patients and NCs. However, more research is needed to confirm whether these changes are also present in other structural and functional networks.

This study has some limitations. First, the results could not survive when we adopted Family-wise Error (FWE) or False Discovery Rate (FDR) to adjust for multiple comparisons. To increase the statistical power, a larger sample size is needed. Second, given that most relevant studies employed AAL-90 template to define the nodes, we also used the same template in order to allow better comparison with previous results. It should be note that AAL-90 atlas is a structure template which do not include the cerebellum, and it may not be appropriate for studying brain functions. Future studies should therefore validate these findings by using other functional parcellations such as Power-264 (Power et al., 2011). Third, partial volume correction was not used in this study, therefore the results may in part reflect structural variation due to DM or other etiology. Fourth, because recent evidence suggests that anticorrelation might have special importance for understanding brain dysfunction in mental illness (Whitfield-Gabrieli and Ford, 2012; Buckner et al., 2013), we used absolute threshold value to binarize the matrix and did not separate the anticorrelation in our study. However, owing to the way PET data are typically normalized (dividing regional values by the global values), the anticorrelation is likely to be introduced (Murphy et al., 2009), which may make the complete interpretation of the sign of correlation more difficult. Thus, new strategies for processing PET data is required to be further study to mitigate the specific issue of normalization. Lastly, the lack of objective and specific neurocognitive assessment limited our interpretation of the results. Thus, further work should be conducted to investigate whether the functional network abnormalities is associated with the trajectories from normal cognition to dementia in diabetes, even in prediabetic stages, which will help to increase understanding of the DM-related cognitive decline.

## Conclusion

We have investigated the topological properties of human brain functional networks in DM patients using FDG PET and graph theory analysis. Our result indicated that diabetic brain was related to disrupted topological organization in the functional PET network, which was also compatible with a recent study of brain structural networks. Specifically, we found that the DM patients had significantly reduced nodal centrality of several brain regions. Thus, our findings provided the functional evidence for abnormalities of brain networks in DM.

## Author contributions

Conceived and designed the experiments: XQ, YZ. Performed the experiments: XQ, HF, and DJ. Analyzed the data: XQ, HF, and DJ. Wrote the paper: XQ, YZ.

### Conflict of interest statement

The authors declare that the research was conducted in the absence of any commercial or financial relationships that could be construed as a potential conflict of interest.
